# Comparative efficacy of single-inhaler triple therapies for COPD: A protocol for systematic review and network meta-analysis

**DOI:** 10.1371/journal.pone.0255545

**Published:** 2021-08-05

**Authors:** Yixuan Jiang, Hao Hu, Siu-wai Leung

**Affiliations:** 1 State Key Laboratory of Quality Research in Chinese Medicine, Institute of Chinese Medical Sciences, University of Macau, Taipa, Macau SAR, China; 2 Shenzhen Institute of Artificial Intelligence and Robotics for Society, Shenzhen, China; 3 Edinburgh Bayes Centre for AI Research in Shenzhen, College of Science and Engineering, University of Edinburgh, Edinburgh, United Kingdom; Mayo Clinic Minnesota, UNITED STATES

## Abstract

**Introduction:**

2021 Global Initiative for Chronic Obstructive Lung Disease (GOLD) Reports recommends that patients with clinically significant symptoms and exacerbations of chronic obstructive pulmonary disease (COPD) should escalate to triple therapy, a combined use of inhaled corticosteroids (ICS), long-acting muscarinic antagonists (LAMA) and long-acting b2-agonists (LABA)(ICS/LAMA/LABA). Triple therapy in fixed-dose combinations (FDCs), i.e., combining ICS, LABA with LAMA and administrating by a single inhalation device, has appeared in recent years. This study aims to compare the efficacy of triple therapy in FDCs in treating patients with moderate to severe COPD.

**Methods and analyses:**

Literature search will be conducted on PubMed, Embase and Web of science, according to pre-specified and corresponding search strategies, for relevant reports published since the inception dates of the databases. Randomised controlled trials (RCT) which compared the triple therapy in FDCs with other pharmacological therapies will be included. The Cochrane risk of bias assessment tool (RoB 2) will be used to assess the RCT quality. The outcomes will be analyzed as rate ratios and mean differences under a random-effects model in a frequentist network meta-analysis (NMA). Additional statistical analyses including subgroup analysis, sensitivity analysis, and publication bias analysis will be performed to assess the evidential heterogeneity and robustness. The strength of evidence from the NMA will be evaluated with the Grading of Recommendation, Assessment, Development and Evaluation (GRADE) methods.

**Ethics and dissemination:**

No ethics approval is required as this systematic review and network meta-analysis do not collect confidential personal data and do not carry out interventions in treating patients.

**Protocol registration number:**

CRD42021240823.

## Introduction

Chronic obstructive pulmonary disease (COPD) is a type of obstructive lung diseases characterised by persistent respiratory symptoms especially airflow limitation [1]. Its main symptoms include shortness of breath and cough with sputum production [1]. Tobacco smoking is the most common cause of COPD, followed by air pollution and genetics [2]. Significant comorbidities may have an impact on morbidity and mortality [3]. The latest statistical analyses indicated that there were about 174.5 million [4] COPD cases and 3.2 million deaths [5] worldwide as of 2015. These numbers are likely to increase in coming years with the increasing smoking prevalence and aging populations in many countries [6].

COPD is a progressive disease. Pharmacotherapy of COPD improves common symptoms, exacerbations, general health, and exercise tolerance [7]. Global Initiative for Chronic Obstructive Lung Disease (2021 REPORT) recommended that the COPD patients with persistent symptoms or history of exacerbations should be treated with combined long-acting muscarinic antagonists (LAMA) and long-acting b2-agonists (LABA)(LAMA/LABA), or combined LABA and inhaled corticosteroids (ICS)(LABA/ICS). The patients who are on LABA/LAMA therapy or LABA/ICS therapy but still suffer from persistent breathlessness, exercise limitation, or further exacerbations should be escalated to triple (i.e., LABA/LAMA/ICS) therapy [1, 8, 9]. As of March 2021, the triple therapies in FDCs available on the market are listed in Table 1.

**Table 1:** Summary of marketed triple therapies in FDCs.

Trade name	Manufacturer	Ingredients	Inhaler Type
Trimbow^®^ Spray	Chiesi	beclomethasone dipropionate, glycopyrronium and formoterol fumarate (BDP/FF/G)	MDI
TRELEGY^®^ ELLIPTA^®^	GlaxoSmithKline	fluticasone furoate, umeclidinium and vilanterol (FF/UMEC/VI)	DPI
Breztri Aerosphere^™^	AstraZeneca	budesonide, glycopyrrolate and formoterol fumarate (BUD/GLY/FF)	MDI

FDC, fixed-dose combination; MDI, metered dose inhaler; DPI, dry powder inhalers.

There are three methods of administration for triple therapy: (a) three single drugs to be administrated with three separate inhalation devices, (b) administration with two inhalation devices to combine ICS/LABA with a LAMA (designated as “ICS/LABA + LAMA”) or LABA/LAMA with ICS (designated as “LABA/LAMA + ICS”), and (c) combining three drugs ICS, LABA, and LAMA in a single inhalation device [10]. Administration by method (a) and method (b) is called “open triple combination therapy”, while method (c) is called “triple therapy in a fixed-dose combination (FDC)” [10]. It was suggested that “LABA/LAMA + ICS” is more suitable for patients in COPD, while “ICS/LABA + LAMA” is more suitable for the patients suffering from asthma [11]. López [10] recommended that the administration by method (c) is a simple, efficient, and potentially cost-effective treatment, better than the methods (a) and (b). However, as yet no large-scale RCTs nor network meta-analyses of RCTs have been conducted to confirm their findings.

Published systematic reviews [12, 13] and pairwise meta-analyses [14–17] on previous RCTs [18–25] claimed that triple therapy in FDCs, e.g., BDP/FF/G, FF/UMEC/VI and BUD/GLY/FF, could be better than monotherapy and dual therapy in improving lung function and quality of life. The only network meta-analysis [26] included merely seven [18–23, 27] RCTs with 8762 participants on triple therapy in FDCs well before August 2019. It left out the 4987 participants and new findings from recent large-scale RCTs [24, 25] on triple therapy. It is thus warranted to conduct a new network meta-analysis like the present study to investigate whether the new findings of the recent large-scale RCTs would support or invalidate the past recommendations.

Even if there were no uncovered large-scale RCTs, the past systematic reviews and meta-analyses should be revisited as they were not fully (albeit claimed to be) compliant with the PRISMA 2009 statement [28], let alone PRISMA-NMA statement [29] and PRISMA 2020 statement [30]. They did not meet essential requirements for meta-analysis, e.g., protocol registration [13, 16, 17], assessment of the study quality by Cochrane risk of bias tool version 2 (RoB 2) [13, 31], evidence strength evaluation by the Grades of Recommendations, Assessment, Development and Evaluation (GRADE) [12, 13, 16, 17, 32], and statistical analyses such as subgroup analysis and publication bias [12–14, 16, 17]. In addition to PRISMA 2009 [28] and PRISMA 2020 [30], the latest network meta-analysis was not fully compliant with the PRISMA-NMA statement [29] that network meta-analyses should observe.

For the above reasons, this study aims to conduct a PRISMA-NMA compliant and comprehensive NMA on the RCTs comparing the efficacies of various triple therapies in fixed-dose combinations in treating the patients suffering from moderate to severe COPD.

## Materials and methods

The protocol of this systematic review and network meta-analysis will be reported in accordance with the Preferred Reporting Item for Systematic Review and Meta-analysis Protocols (PRISMA-P) [33]. The completed study will be compliant with the PRISMA 2020 [30] and PRISMA-NMA [29].

### Eligibility criteria

Detailed eligibility criteria were developed following the PICOS heuristic, i.e., Population/participants, Intervention, Comparators, Outcomes, and Study design (PICOS) [34], as summarised in Table 2.

**Table 2:** Summary of eligibility criteria.

Participants	Participants aged over 40 years of any gender or region with moderate to severe COPD (FEV_1_ predicted 30% to 80%)
Interventions	Triple therapies in fixed-dose combinations
Comparators	Triple therapies in open combinations, dual therapies, monotherapies, placebo
Outcomes	Trough FEV_1_, rate of moderate or severe exacerbations and SGRQ score
Study Design	Randomised controlled trials of 10 weeks duration or more

COPD, chronic obstructive pulmonary disease; FEV_1_, Forced Expiratory Volume in the first second; SGRQ, St George’s Respiratory Questionnaire.

Eligible participants must be aged over 40 years and suffering from moderate to severe COPD (defined by Forced Expiratory Volume in the first second [FEV_1_] less than 80% and more than 30%), regardless of their genders, regions, or any non-specified baseline characteristics. Asthma patients will be excluded. Eligible interventions in the RCTs are treatments with at least one of the fixed triple therapies, e.g., BDP/FF/G, FF/UMEC/VI, and BUD/GLY/FF, against controls such as triple therapy in open combination, dual therapy (ICS/LABA and LAMA/LABA), monotherapy (ICS, LAMA and LABA), and placebo. Eligible outcome measures in the RCTs include changes from baseline in trough FEV_1_, rates of moderate or severe exacerbations, and total scores of St George’s Respiratory Questionnaire (SGRQ). In addition to the above criteria, eligible study design must be RCTs with follow-up periods of 10 weeks or more. Observational cohort studies, case-control studies, case reports, and reviews will be excluded.

### Literature search

Two investigators will search for relevant RCT reports from three electronic databases (PubMed, Web of Science, and Embase) for relevant publications starting from the inception dates of databases. References cited in the included publications will be manually searched to check for additional relevant RCT reports. The terms “COPD”, drug names, “RCT” will be used as keywords to search titles or abstracts. Search strategies for PubMed, Web of Science, and Embase are as shown in S1 Appendix.

### Study selection

**Fig 1:**
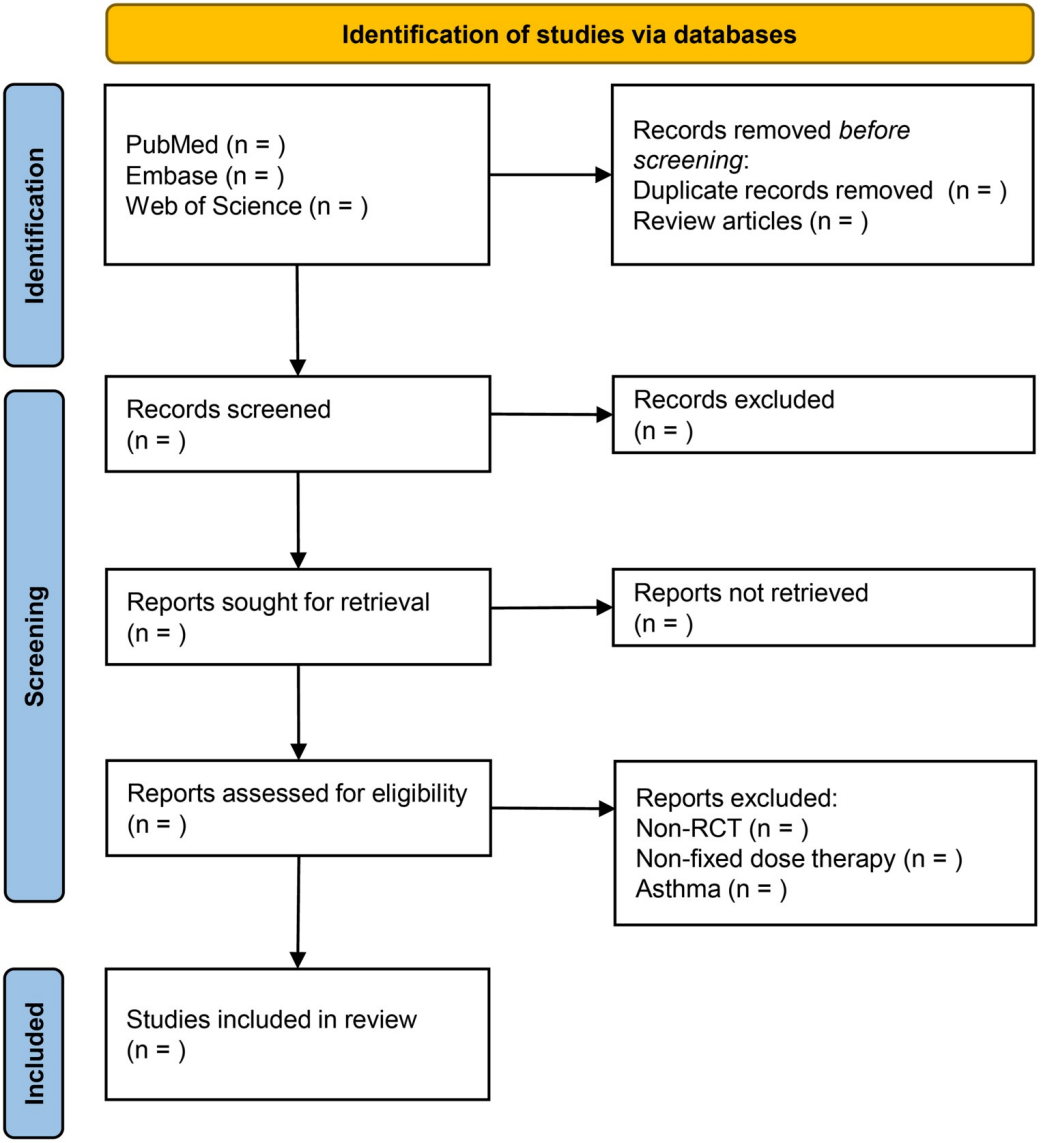
Flow chart of the study selection. RCT, randomised controlled trial.

All retrieved records will be imported into EndNote (X8) for data management. Duplicates will be removed. Two investigators will independently screen the retrieved records according to the eligibility criteria. Disagreements between investigators will be resolved by discussion with the principal investigator. The process of study selection will be shown in a PRISMA-compliant [30] flowchart as exemplified in Fig 1.

### Data management

Two investigators will independently extract the data from the included full reports using a standard template. Any discrepancies between two sets of data will be resolved by discussion. Unresolved discrepancies will be brought up to the principal investigator for final decision. Specific data points of interest that were only presented in graphs will be extracted using WebPlotDigitizer [35].

Specific information of the following items will be extracted: (a) study characteristics including author names, publication years, follow-up durations, etc., (b) participant/population characteristics including sample sizes, mean ages, proportions of males, proportions of smokers, proportions of the participants with exacerbation history, FEV_1_ predicted and inclusion criteria, (c) interventions and comparators including drug names, doses, regimens, and devices, and (d) outcomes including the definitions of exacerbation and time points of assessment.

Changes from baseline in trough FEV_1_ are the common endpoints to measure lung function among the RCTs are the main outcome. St George’s Respiratory Questionnaire (SGRQ) total scores, as a measure of health-related quality of life (HRQoL), and rates of moderate or severe exacerbations, are additional outcomes. For changes from baseline in trough FEV_1_ and SGRQ total scores, observation time points of 12 and 24 weeks will be chosen. In the absence of 24-week data, data within a 2-week range for each time point of interest will be allowed (i.e., between 24 and 22 weeks for the 26-week time point). Rates of moderate or severe exacerbations up to week 24 will be evaluated.

### Risk of bias assessment

The quality of eligible studies will be evaluated independently by two investigators according to Version 2 of the Cochrane tool for assessing risk of bias in randomised trials (RoB 2) [31] for assessing risk of bias (Table 3). RoB 2 will assess five domains, including randomisation process, deviations from intended interventions, missing outcome data, measurement of the outcome, selection of the reported result. Each domain will be judged as “low risk of bias,” “some concerns,” or “high risk of bias” by the answers (i.e., yes / probably yes / probably no / no / no information) to the respective signalling questions. Any discrepancy and disagreement between investigators will be resolved by discussion among investigators or consulting the principal investigator.

**Table 3:** RCT quality assessment according to the Cochrane risk-of-bias assessment tool (version 2).

	RCT 1	RCT 2	RCT 3	RCT 4	RCT 5
Randomisation selection					
Deviations from intended interventions					
Missing data					
Measurement of the outcome					
Selection of the reported result					
**Overall bias**					

RCT, randomised controlled trial.

### Meta-analysis

A network meta-analysis based on a frequentist framework [36] will be conducted with a random effects model due to the expected heterogeneity, using the package ‘netmeta’ under the R environment version 4.0.4. [37, 38] Continuous outcomes will be represented by mean difference (MD) and 95% confidence intervals (CIs). Rate ratios (RR) and 95% CI will represent the dichotomous outcomes. P-scores will be used to rank the treatments according to individual outcome measures [39]. If sufficient data are available, subgroup analysis based on study characteristics, such as mean ages, history of exacerbation, and lung function, will be conducted to explain the heterogeneity. Sensitivity analysis will be also conducted, e.g., excluding the studies of poor quality or with a high risk of bias.

### Publication bias

Funnel plots [40] will be generated with the package ‘metafor’ under the R environment version 4.0.4 [38, 41, 42] to visualise potential publication bias. Begg’s method [43] and Egger’s method [44] will be carried out to quantify the potential publication bias and its statistical significance.

### Strength of evidence

Two investigators will independently evaluate the quality of evidence for outcomes in accordance with the GRADE [32] by the software GRADEpro [45]. The strength of evidence will be reported as high, moderate, low, or very low, according to the five domains, i.e., study limitations, inconsistency, indirectness, imprecision, and publication bias.

## Discussion

This network meta-analysis will summarise the available evidence from randomised controlled trials compared the efficacy of triple therapy in fixed-dose combinations including BDP/FF/G, FF/UMEC/VI and BUD/GLY/FF MDI in treating patients suffering from moderate to severe COPD. To compensate the inadequacy of the past meta-analyses, we will conduct subgroup and sensitivity analyses based on the study quality of the included RCTs according to the RoB 2 [31], to reveal the potential impact of the study quality of RCTs on the overall results. The evaluation for the strength of evidence, particularly according to the GRADE approach, that were left out from the past network meta-analyses will be assessed for the first time. It is anticipated that the present study provides a PRISMA-NMA-compliant network meta-analysis together with required subgroup analysis, sensitivity analysis, and publication bias analysis to reveal the robustness and strength of evidence for comparative efficacies of available triple therapies in FDCs. This study will inform physicians, patients and their families, and guideline developers of the best available evidence for their decision making.

## Supporting information

S1 Checklist PRISMA-P checklist. (DOCX)

S1 Appendix Search strategies. (DOCX)
