# Supplementary material for: Comparative efficacy of single-inhaler triple therapies for COPD: A protocol for systematic review and network meta-analysis
Source: PLoS One. 2021 Aug 5;16(8):e0255545. doi: 10.1371/journal.pone.0255545 (PMC8341520; doi:10.1371/journal.pone.0255545)
Supplement: S1 Appendix — (DOCX) [file pone.0255545.s002.docx]

**S1 Appendix: PubMed and Web of Science Embase search strategies.**

| **PubMed search strategy** |
| --- |
| (("pulmonary disease, chronic obstructive"[MeSH Terms] OR ("pulmonary"[All Fields] AND "disease"[All Fields] AND "chronic"[All Fields] AND "obstructive"[All Fields]) OR "chronic obstructive pulmonary disease"[All Fields] OR "copd"[All Fields])) AND ((("beclomethasone"[MeSH Terms] OR "beclomethasone"[All Fields] OR ("beclomethasone"[All Fields] AND "dipropionate"[All Fields]) OR "beclomethasone dipropionate"[All Fields]) AND ("glycopyrrolate"[MeSH Terms] OR "glycopyrrolate"[All Fields] OR ("glycopyrronium"[All Fields]) AND ("formoterol fumarate"[MeSH Terms] OR ("formoterol"[All Fields] AND "fumarate"[All Fields]) OR "formoterol fumarate"[All Fields])) OR (("fluticasone furoate"[Supplementary Concept] OR "fluticasone furoate"[All Fields]) AND ("gsk573719"[Supplementary Concept] OR "gsk573719"[All Fields] OR "umeclidinium"[All Fields]) AND ("vilanterol"[Supplementary Concept] OR "vilanterol"[All Fields])) OR (("budesonid"[All Fields] OR "budesonide"[MeSH Terms] OR "budesonide"[All Fields] OR "budesonide s"[All Fields]) AND ("glycopyrrolate"[MeSH Terms] OR "glycopyrrolate"[All Fields]) AND ("formoterol fumarate"[MeSH Terms] OR ("formoterol"[All Fields] AND "fumarate"[All Fields]) OR "formoterol fumarate"[All Fields]))) AND ("randomized controlled trial"[Publication Type] OR "randomized controlled trials as topic"[MeSH Terms] OR "randomised controlled trial"[All Fields] OR "randomized controlled trial"[All Fields]) |
| **Web of Science search strategy** |
| (TS=(chronic obstructive pulmonary disease) OR TS=(chronic obstructive lung disease)) AND ((TS=(beclomethasone dipropionate) AND TS=(glycopyrronium OR glycopyrrolate) AND TS=(formoterol fumarate)) OR (TS = (fluticasone furoate) AND TS = (gsk573719 OR umeclidinium) AND TS = (vilanterol)) OR (TS=(budesonide) AND TS=(glycopyrrolate) AND TS=(formoterol fumarate)) AND (TS=(randomized controlled trial)OR TS=(randomized controlled trial))) |
| **Embase search strategy** |
| (('chronic obstructive lung disease'/exp OR 'chronic obstructive lung disease' OR (chronic AND obstructive AND ('lung'/exp OR lung) AND ('disease'/exp OR disease))) AND ('chronic obstructive pulmonary disease'/exp OR 'chronic obstructive pulmonary disease' OR (chronic AND obstructive AND pulmonary AND ('disease'/exp OR disease)))) AND ((('beclomethasone dipropionate'/exp OR 'beclomethasone dipropionate' OR (('beclomethasone'/exp OR beclomethasone) AND dipropionate)) AND ('glycopyrronium'/exp OR 'glycopyrronium' OR 'glycopyrrolate'/exp OR glycopyrrolate) AND ('formoterol fumarate'/exp OR 'formoterol fumarate' OR (('formoterol'/exp OR formoterol) AND ('fumarate'/exp OR fumarate)))) OR (('fluticasone furoate'/exp OR 'fluticasone furoate' OR (('fluticasone'/exp OR fluticasone) AND furoate)) AND ('umeclidinium'/exp OR umeclidinium) AND ('vilanterol'/exp OR vilanterol OR 'gsk573719'/exp OR gsk573719)) OR (('budesonide'/exp OR budesonide) AND ('glycopyrrolate'/exp OR glycopyrrolate) AND ('formoterol fumarate'/exp OR 'formoterol fumarate' OR (('formoterol'/exp OR formoterol) AND ('fumarate'/exp OR fumarate))))) AND ('randomized controlled trial'/exp OR 'randomized controlled trial' OR (randomized AND controlled AND ('trial'/exp OR trial))) |
